# Introduction: luteinizing hormone for life’s journey

**DOI:** 10.1186/s12958-025-01357-4

**Published:** 2025-04-17

**Authors:** Carlo Alviggi, Sandro C. Esteves, Robert Fischer, Peter Humaidan

**Affiliations:** 1 Department of Public Health, University of Naples Federico II, Naples, Italy; 2 Andrology and Human Reproduction Clinic, Campinas, Brazil; 3 Department of Surgery (Division of Urology), University of Campinas (UNICAMP), Campinas, Brazil; 4 Faculty of Health, Department of Clinical Medicine, Aarhus University, Aarhus, Denmark; 5 MVZ Fertility Center Hamburg GmbH, Hamburg, Germany; 6 Skive Regional Hospital, Fertility Unit, Skive, Denmark

## Background

Since its identification in 1929, the immunomodulatory functions of the gonadotropin luteinizing hormone (LH) [1] have been of key scientific and clinical interest, with many dedicated conferences for experts in medically assisted reproduction (MAR) to discuss and exchange ideas. One of these was the 3rd World Conference on Luteinizing Hormone in ART: Applying Technologies Learning from Nature (Naples, Italy; May 2022).

With the development of recombinant preparations, which enabled the roles of follicle-stimulating hormone (FSH) and LH in human fertility to be investigated separately [2], LH supplementation has been explored in normal and suboptimal responders to ovarian stimulation (OS). A key aim of such research has been to improve MAR outcomes in subgroups of ‘low-prognosis’ patients, such as women included in the Patient-Oriented Strategies Encompassing IndividualizeD Oocyte Number (POSEIDON) classification system [3, 4], especially those of advanced reproductive age [3, 5]. Other areas of particular interest include better understanding the molecular mechanism driving the LH dependence in early folliculogenesis, evaluating the long-term outcomes of IVF treatments on the development of resultant offspring, and the role of LH in treating male infertility.

There been considerable advances in the use of LH supplementation in the field of MAR, such as approaches to LH-based pharmacogenomics; but there remain challenges and opportunities for further development of new, patient-optimized treatment regimens and applications.

### Topics

The main focus of the included papers in this supplement is to review evidence for the role of LH in advancing MAR and improving outcomes in subgroups of low-prognosis patients.

Longo et al. (2025) [6] analyse recent research using animal and human models exploring the role of LH in folliculogenesis, with a focus on possible new clinical applications. Follicular development is complex and even though it is controlled by the gonadotropins, FSH and LH, it is LH that is associated with follicular remodelling, growth and the conclusion of cell division [7]. The authors discuss data from preantral murine follicles cultured in vitro with human (h)-FSH and h-LH, which suggest a relevant role for LH in promoting and accelerating the progression of ovarian follicles throughout folliculogenesis [8]. In addition, some clinical conditions such as hypothalamic amenorrhoea, pregnancy, hormonal contraception, and long-term use of gonadotropin-releasing hormone (GnRH) analogues (which has been associated with low serum LH levels) may represent in vivo models for exploring the effects of LH depletion on ovarian function. Longo et al. (2025) suggest how clinical applications of these findings may add to the efficacious treatment options available for infertile or subfertile women [6].

Roque and Sunkara (2025) [9] review the outcomes associated with OS, which is a major component of IVF treatment, to maximise the efficacy and safety of MAR. They evaluate the essential role of OS in IVF and the various factors that influence its success which include the woman’s age, body mass index, ovarian reserve, infertility diagnosis, lifestyle factors and genetics. The safety events that can be associated with OS – especially iatrogenic ovarian hyperstimulation syndrome (OHSS), a life-threatening condition caused by an excessive response to OS – are also discussed. The range of clinical, preclinical, laboratory and safety outcomes summarized by Roque and Sunkara may help to quantify OS and provide indicators of its success. However, the authors are keen to emphasize that there is still ‘room for improvement’ in OS strategies. Future developments, such as improving the accuracy of OS prediction or success rates and obtaining clinical data to assess long-term effects of MAR on the offspring, are warranted.

Although LH is critical for successful folliculogenesis, the value of its use during OS is unclear. For their narrative review, Alviggi et al. (2025) [10] completed a literature search (no restrictions to language or years of publication) to investigate whether recombinant human LH (r-hLH) activity supplementation in OS could improve MAR outcomes in specific infertile populations undergoing treatment. Their research supports the use of r-hLH for OS in four specific subgroups of women undergoing IVF: those aligned with the highest Poor Responder Outcome Prediction (PROsPeR) score; those in POSEIDON groups 1 and 2; those ≤ 40 years of age (POSEIDON groups 2 and 4); and those with hypogonadotropic hypogonadism (HH), who are characterized by having very low endogenous gonadotropin levels.

Conforti et al. (2025) [11] summarize current approaches to LH-based pharmacogenetic treatment (i.e., medications prescribed based on the individual’s genetic profile). Their literature review (no restrictions to language or years of publication) compiled evidence from in vitro (molecular pathways in LH signalling) and in vivo studies; it also considers the impact of LH and LH/choriogonadotropin receptor (LHCGR) genetic variants on signal transduction during folliculogenesis, and the clinical effects of *LHB* and LHCGR genetic variants. The authors found that very few studies within the field of MAR have taken a pharmacogenomic approach, and those approaches based on *LHB* and/or *LHCGR* genotypes seem underresearched. Even the promising cumulative effect between LH and LHCGR single-nucleotide polymorphisms are yet to be supported in the randomized clinical trial (RCT) arena. Thus, the authors conclude that it is not yet possible to suggest a pharmacogenomic approach in OS.

Esteves and Humaidan (2025) [12] highlight the critical role of LH in spermatogenesis and the potential role of LH-containing drugs in the treatment of male infertility. Spermatogenesis is primarily regulated by FSH and LH, the latter to stimulate intratesticular testosterone (ITT) production from Leydig cells; adequate ITT levels are required for spermiogenesis and in combination with FSH, ITT contributes to sperm cell proliferation [13]. Here, Esteves and Humaidan (2025) examine the data regarding the effectiveness of LH-containing drugs in treating specific male infertility conditions, particularly HH and non-obstructive azoospermia (NOA). Gonadotropin treatment that involves LH activity may enable a greater number of infertile men to achieve biological fatherhood than has previously been possible. However, the authors highlight that the efficacy of LH-containing gonadotropins in treating male infertility requires further validation through large-scale, well-designed RCTs and prospective observational studies. These investigations should compare gonadotropin therapy versus no treatment, the best treatment protocols, and therapy duration.

Orvieto (2025) [14] evaluates studies relating to the ways that final follicular maturation may be triggered, and how each approach may be tailored to specific patient subgroups. In particular, he discusses the ‘dual trigger’ regimen (gonadotropin-releasing hormone agonist [GnRHa] plus human chorionic gonadotropin [hCG]) for controlled ovarian hyperstimulation (COH) and cites the positive outcomes (e.g., excellent-quality embryos and increasing rates of implantation, clinical pregnancy and live birth) achieved in normal responders 45–37 h before oocyte retrieval in prospective randomized and retrospective cohort studies. Furthermore, Orvieto examines evidence for a dual trigger regimen (34 h before oocyte pick-up [OPU]) in poor responders, as defined by the Bologna criteria [15]. A ‘double-trigger’ approach (GnRHa plus standard hCG added 40 and 34 h before OPU, respectively) is also discussed in two groups of patients showing abnormal final follicular maturation despite normal response to COH; this approach led to an increased number of top-quality embryos compared with hCG-only trigger cycles [16].

Following the theme that MAR should be personalized, Vaiarelli et al. (2025) [17] assessed evidence for the double-stimulation protocol, back-to-back in the same ovarian cycle (DuoStim), to rescue anovulatory waves. Several DuoStim protocols have been proposed, but they vary considerably across studies; no one protocol has demonstrated superiority over others. However, data show that these protocols may be efficacious in women of advanced reproductive age or poor/suboptimal responders, as they enable more euploid blastocysts to be obtained per cycle and reduce the time required to obtain embryos. Here, Vaiarelli et al. (2025) acknowledge that more data are required from RCTs and real-life experiences, involving DuoStim, especially to verify the reductions in time to pregnancy and dropout rate of patients.

## Discussion and conclusion

The papers included in this supplement highlight current research trends and areas for future investigation in LH and MAR. They discuss the latest data in several areas of key research investigating the use of LH in infertility. It is envisioned that these data will have a substantial impact on the direction and application of LH in the future MAR domain.

## Data Availability

Not applicable.

## Abbreviations

ART: Assisted reproductive technology
COH: Controlled ovarian hyperstimulation
h: Human
FSH: Follicle-stimulating hormone
GnRH(a): Gonadotropin-releasing hormone (agonist)
hCG: Human chorionic gonadotropin
HH: Hypogonadotropic hypogonadism
ITT: Intratesticular testosterone
IVF: In vitro fertilization
LH: Luteinizing hormone
LHCGR: LH/choriogonadotropin receptor
MAR: Medically assisted reproduction
NOA: Non-obstructive azoospermia
OHSS: Ovarian hyperstimulation syndrome
OPU: Oocyte pick-up
OS: Ovarian stimulation
POSEIDON: Patient-Oriented Strategies Encompassing IndividualizeD Oocyte Number
PROsPER: Poor Responder Outcome Prediction
r: Recombinant
RCT: Randomized controlled trial
